# Supplementary material for: Lasy-Seq: a high-throughput library preparation method for RNA-Seq and its application in the analysis of plant responses to fluctuating temperatures
Source: Sci Rep. 2019 May 8;9:7091. doi: 10.1038/s41598-019-43600-0 (PMC6506593; doi:10.1038/s41598-019-43600-0)
Supplement: Supplementary file 1 — Supplementary information [file 41598_2019_43600_MOESM1_ESM.pdf]

Supplementary Information

for

Lasy-Seq: a high-throughput library preparation method for RNA-Seq and its application in the analysis of plant responses to fluctuating temperatures

Mari Kamitani, Makoto Kashima, Ayumi Tezuka & Atsushi J. Nagano*

Correspondence to: anagano@agr.ryukoku.ac.jp

This PDF file includes:

Supplementary Fig. S1 Primers used in the present study.

Supplementary Fig. S2 Method for calculation of false-assignment rates

Supplementary Fig. S3 Overview of the analysis of RNA-Seq data

Supplementary note 1 The protocol of Lasy-Seq with detailed notes

Supplementary note 2 R and Python Script for RNA-Seq analysis

Supplementary Data File includes:

Supplementary table S1 Mapping rate of samples used in this study

Supplementary table S2 Summary of the false-assignment rates reported by previous studies

Supplementary table S3 List of genes significantly correlated to temperature on each day

Supplementary table S4 GO terms significantly-enriched for each time points

Supplementary table S5 Information on the samples collected in this study (n = 45)

(A)

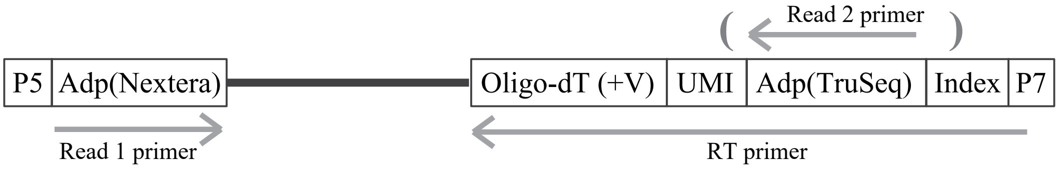

(B)

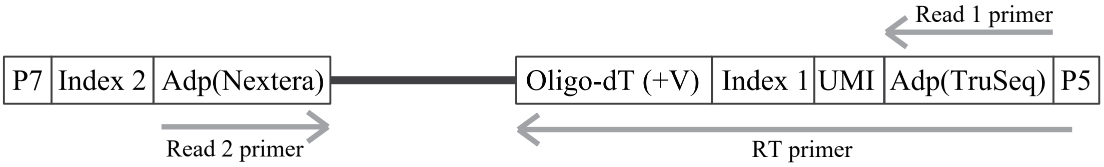

(C)

Primer	Sequence(5'-3')
SR RT-primer (SR)	CAGAAGACGGCATACGAGATxxxxxxxx<br>GTGACTGGAGTTCAGACGTGTGCTCTTCCGATCNNNNNNTTTTTTTTTTTTTTTTTTV
SR PCR forward-primer (SR)	CAAGCAGAAGACGGCATACGAGAT (for 5' part of every RT primers)
SR PCR reverse-primer (SR)	AATGATACGGCGACCACCGAGATCT(2nd index can be inserted here)ACACTCGTCGGCAGCGTC<br>(for the Nextera tagmentation adapter sequence)
PE60 RT-primer (PE)	CCCTACACGACGCTCTTCCGATCTNNNNNNxxxxxxxxxxTTTTTTTTTTTTTTTTTTVN
PE78 RT-primer (PE)	CCCTACACGACGCTCTTCCGATCTNNNNNNNNNNNNxxxxxxxxxxTTTTTTTTTTTTTTTTTTTT<br>TTTTTTTTTVN
PE PCR forward-primer (PE)	AATGATACGGCGACCACCGAGATCTACACACACTCTTTCCCTACACGACGCTCTTCCGATCT<br>(for 5' part of every RT primers)
PE PCR reverse-primer (PE)	Mixture of 8 primers with different index sequences.<br>CAAGCAGAAGACGGCATACGAGAT********GTCTCGTGGGCTCGG<br>(example ******** : ccgagggt, tcagctgt, cttgctgt, gtagacgc, agtgacac, ggtaattc, ttggccca, ctgtgttg)<br>(for the Nextera tagmentation adapter sequence)

Supplementary figure 1　Primers used in the present study.

(A) Schematic drawing of the library for the single-read sequencing (SR). P7 and P5 indicates sequences which are required for the hybridization to the flowcell (Illumina). Adp(TruSeq) and Adp(Nextera) means the adapter sequences used in the TruSeq and Nextera kit, respectively. **(B)** Schematic drawing of the library for the paired-end sequencing. In paired-end sequencing, quality of the latter part of the read 1 becomes low because of sequencing the polyA sequences, only index1 and UMI in read1 and read 2 sequences can be used for the analysis. **(C)** List of the sequences of the primers used in the present study. The "x" and "N" indicate index sequences and random bases (UMI). "V" meands bases of either "A" , "C" or "G" . RT-indexing primer with 78mer in length was constructed depending on a previous study (PE78 RT-primer, Cao et al. 2017). Shorter RT primer (PE60 RT-primer) were designed to save cost of preparing primers. Diversity of index sequences were required for Illumina platforms (PE PCR reverse-primer).

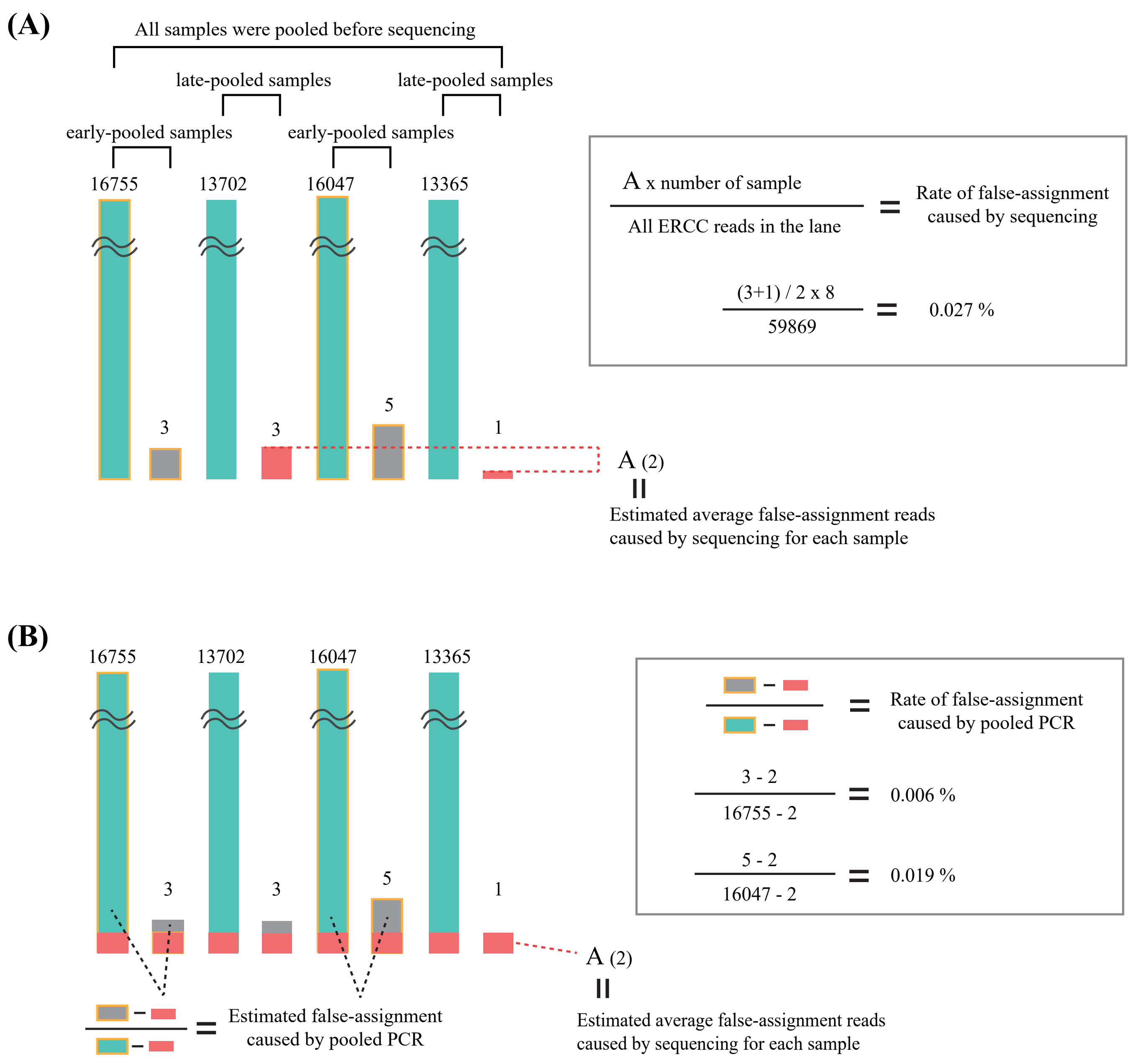

Supplementary figure 2 Method for calculation of false-assignment rates

Method to calculate false-assignment rate caused by sequencing and pooled PCR used in the present study. (A) Method for calculation of false-assignment rate caused by sequencing. (B) Method for calculation of false-assignment rate caused by pooled PCR. Detaile of each sample was indicated in Fig. 2 and Materials and Methods section.

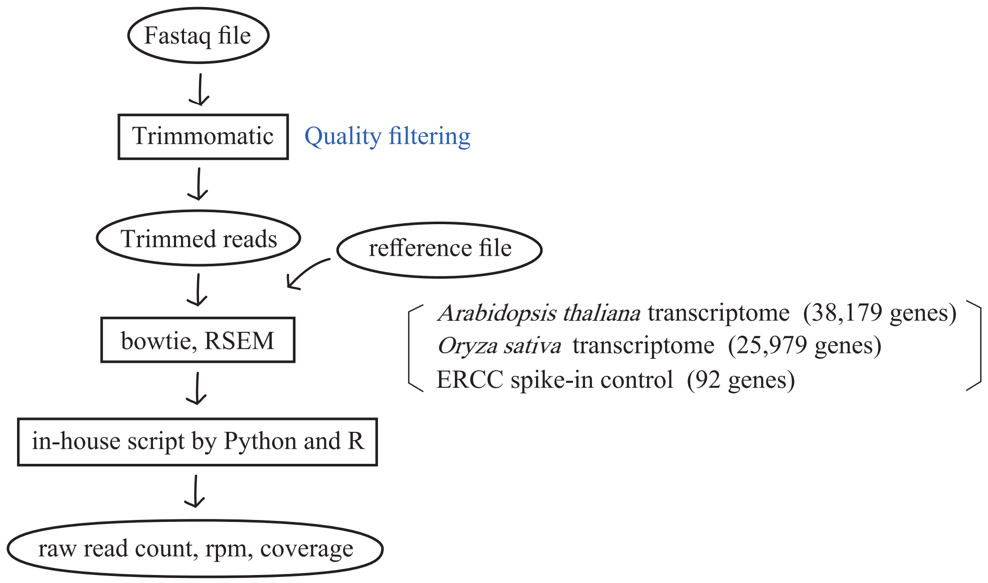

Supplementary figure 3 Overview of the analysis of RNA-Seq data.

The FASTQ files obtained from RNA-Seq were filtered by trimomatic. Then the trimmed reads were mapped onto the plant transcriptome sequence and ERCC sequences. The produced bam files were analyzed by RSEM to calculate expected read count and in-house script by Python and R to calculate rpm and the coverage. The script was provided in supplementary note 2. Detailed protocols are described in our previous study (Kamitani et al. 2016 FEMS Microbial Ecology).

Supplementary note 1: The protocol of Lasy-Seq with detailed notes

Things to know before you start

- ✓ This is a protocol for high-throughput analysis. For a smaller number of samples, optimization is required for the input-amount of RNA and volume of elution-solution in each reaction.

- ✓ This protocol does not require an mRNA-enrichment step. The RT reaction is started from total RNA, after second strand synthesis, RNase treatment is required to remove the large amounts of RNA.

- ✓ We usually conduct single-read sequencing for the quantification of gene expression. Paired-end sequencing can also be used for more precision analysis, because information of UMI is available.

- ✓ The concentration of the libraries produced with Lasy-Seq are sometimes over estimated. In such cases, smaller inputs of libraries into sequencing than Illumina recommends improves results.

This protocol will be updated at the following website as required.

https://sites.google.com/view/lasy-seq/home

1. Reverse Transcription

1-1. Prepare total RNA samples in the PCR plate.

	For each sample	Master mix (for example, *n*=96)	
➢ RNA 500 ng/sample	X μL		
1-2. Assemble the master mix as follows.	For each sample	If using 5 μL of 100 ng/μL RNA,	
➢ 5X Superscript IV First-Strand Buffer	4.0 μL	384 μL	Dispense 14 μL of master mix to each sample
➢ RNasin Plus	0.5 μL	48 μL	
➢ DTT (100 mM)	2.0 μL	192 μL	
➢ dNTP (25 mM each)	0.4 μL	38.4 μL	
➢ SuperScript IV reverse transcriptase	0.1 μL	9.6 μL	
➢ Nuclease-free water	12 - X μL	672 μL	

1-3. Add 19- X μL of master mix to each RNA sample.

1-4. Add 1μL of 2μM SE RT-primer (with index-sequences) to each sample.

1-5. Incubate the plate at 65 °C for 10 min, 80 °C for 15 min and keep the sample at 4°C until the next step.

Note: *Total input RNA (pooled from all samples) is recommended to be more than 10 μg, for example, we successfully constructed libraries with 20 samples of 500 ng RNA for each sample. If the number of samples is small, larger amounts of input RNA should be prepared and vice versa.*

2. Purification

2-1. Pool all of the solutions into a tube.

2-2. Place the RT reaction plate on the one well reservoir (Sasagawa et al. 2018. *Genome Biol*). Centrifuge the plate and collect the solution into the one well reservoir as described in the previous study.

2-3. Collect the solution into a 5mL tube and add 2x Membrane binding solution (Promega) and mix well with voltex mixer. Set the Zymo-Spin Column I (Zymo research) on the Vac-Man Laboratory Vacuum Manifold (Promega) and filtrate the collected solution. Wash twice with 700 μL of wash buffer, mixture of ethanol (final conc. 80%) and Tris-HCl (pH 7.6, final conc. 100 mM). Put the column on a new tube and centrifuge it at 16,000 g at 4 °C for 2 min to remove the wash buffer. Put the column on a new tube and elute the RNA/cDNA hybrid by adding 100 μL of nuclease-free water.

This step can be replaced by AMpure XP beads purification.

3. Second Strand Synthesis

3-1. Assemble the following mix.

➢ RNA/cDNA hybrid	30.0 μL	(Store the rest 70μL at -20°C)
➢ 10x Blue buffer	4.0 μL	
➢ dNTP mix (2. 5 mM each)	2.0 μL	
➢ DTT (100 mM)	1.0 μL	
➢ RNase H	1.0 μL	
➢ DNA polymerase I	2.0 μL	

3-2. Incubate at 16 °C for 2 h and keep at 4°C until the next step.

4. RNase treatment

4-1. Add 2 µL of mixture of 1/1000x RNase T1 (1000 U/µL) and RNase A (10 mg/mL).

4-2. Incubate at 37 °C for 5 min and keep at 4°C until the next step.

4-3. Bind the RNA/cDNA hybrid using 0.8x volume of AMPure XP beads. Purify them following to the manufacturer's instructions. Elute the RNA/cDNA hybrid by adding 10 µL of nuclease-free water.

This purification with AMPure XP beads can be replaced by column purification using Zymo spin column I and Membrane Binding Solution.

5. Quantification of dsDNA

5-1. Quantify the cDNA. (For example with QuantiFluor dsDNA System and Quantus Fluorometer (Promega)).

6. Optimization of tagmentation of dsDNA

6-1. Assemble the following mix.

➢ dsDNA	X µL (Test 3 concentration between 3 and 8 ng, for example, 4ng, 6ng and 8ng)
➢ Tagment DNA buffer (TD)	5.0 µL
➢ Tagment DNA Enzyme (TDE1)	0.5 µL
➢ Nuclease-free water	4.5-X µL

6-2. Incubate at 55 °C for EXACT 5 min.

6-3. Immediately add 50 µL (5x volumes) of DNA Binding Buffer to the cDNA samples. Mix briefly by vortexing

and transfer mixture to a Zymo-Spin Column II set on a new collection tube. Centrifuge at 14,000g at room temperature for 30 sec. Discard the flow-through.

6-4. Add 200 µl DNA Wash Buffer to each column. Centrifuge at 14,000g at room temperature for 30 sec. Repeat this wash step again.

6-5. Transfer the column to a 1.5 ml tube Add 18 µL water directly to the column matrix and incubate at room temperature for 1 minute. Centrifuge at 14,000g at room temperature for 30 sec to elute the dsDNA.

Note: *This optimization step of input-cDNA amounts is necessary, because in libraries with shorter size distributions, sequencing-reads were reached to poly-A sequences at the 3' end of the insert. Libraries distributed from 200 bp to 1500 bp with the average length of 500 bp were efficient enough to decrease the amount of poly-A sequences in data reads.*

The purification step after tagmentation cannot be replaced by purification with AMPure XP beads or NucleoSpin Gel and PCR Clean-up (Takara bio, Japan), in which final yields of the library were largely decreased.

7. Optimization of the number of PCR cycles

7-1. Assemble the following mix.

➢ Tagmented DNA	4.0 µL (for each 3 templates prepared above)

- HiFi HotStart Ready MIX 5.0 µL (for each 3 templates prepared above)
- SE PCR forward-primer (10 µM) 0.5 µL
- SE PCR reverse-primer (10 µM) 0.5 µL

7-2. Add two replicate standards (10 µL) to the wells in the PCR plate following the manufacturer's instructions.

7-3. Incubate at 95°C for 5 min, 30 cycles of 98°C for 20 sec, 60°C for 15 sec, 72°C for 40 sec, followed by 72°C for 3 min and hold at 4 °C until the next step.

7-4. Determine the optimal cycle number by comparing to standards. We usually select the cycle number of 2 or 3 cycles smaller than the cycle at the center of the amplification curve (red-brake line in the figure below).

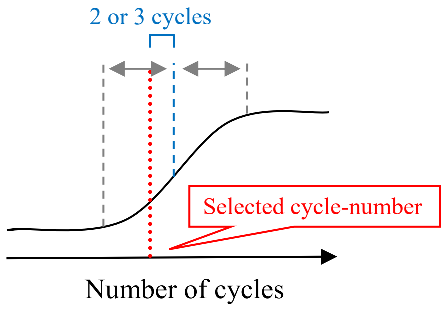

8. Amplification of libraries with optimized PCR cycles

8-1. Assemble the following mix.

- Tagmented DNA 12.0 µL (for each 3 templates prepared above)
- 2x KAPA-HiFi HS Ready MIX 15.0 µL
- SE PCR forward-primer (10 µM) 1.5 µL
- SE PCR reverse-primer (10 µM) 1.5 µL

8-2. Incubate at 95°C for 5 min, optimized cycles of 98°C for 20 sec, 60°C for 15 sec, 72°C for 40 sec followed by 72°C for 3 min and hold at 4 °C until the next step.

8-3. Bind the RNA/cDNA hybrid using same volume of AMPure XP beads. Purify them following the manufacturer's instructions. Elute the RNA/cDNA hybrid by adding 8 µL of nuclease-free water.

9. Analysis on length-distribution of libraries

9-1. Analyze the length-distribution of three purified-libraries by Bioanalyzer with a high sensitivity DNA kit (Agilent Technologies, CA, USA). Determine the amount of cDNA input (ng) that produced libraries distributed from 200bp to 1500bp with the average length of 500bp, and select this as an optimized condition. A representative of recommended library distribution is shown in the figure below.

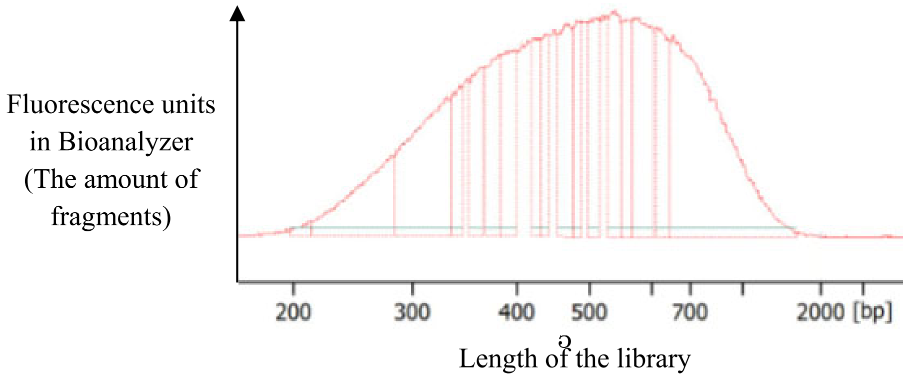

10. Tagmentation of ds-cDNA with optimized input cDNA

10-1. Assemble the following mix **in triplicate**.

- dsDNA X μL (Use optimized amount of input dsDNA)
- Tagment DNA buffer (TD) 5.0 μL
- Tagment DNA Enzyme (TDE1) 0.5 μL
- Nuclease-free water 4.5-X μL

10-2. Incubate at 55 °C for EXACTLY 5 min.

10-3. Immediately add 50 μL (5x volumes) of DNA Binding Buffer to the cDNA samples and mix briefly by vortexing. Pool the three reaction solutions into a tube. Purify the tagmented DNA with a Zymo-Spin Column II as mentioned above. Elute the dsDNA with 54 μL of nuclease-free water.

11. Amplification of libraries with optimized PCR cycles

11-1. Assemble the following mix **in triplicate**.

- Tagmented DNA 16.0 μL (for each 3 templates prepared above)
- 2x KAPA-HiFi HS Ready MIX 20.0 μL (for each 3 templates prepared above)
- SE PCR forward-primer (10 μM) 2.0 μL
- SE PCR reverse-primer (10 μM) 2.0 μL

11-2. Incubate at 95°C for 5minutes, optimized cycle-number of 98°C for 20 sec, 60°C for 15 sec, 72°C for 40 sec, followed by 72°C for 3 min and hold at 4 °C until the next step.

11-3. Bind the RNA/cDNA hybrid using 0.8x volume of AMPure XP beads. Purify them following to the manufacturer's instructions. Elute the RNA/cDNA hybrid by adding 20 μL of nuclease-free water. Pool the triplicated samples.

12. Analysis on length-distribution and concentration of libraries

12-1. Analyze the length-distribution of three purified-libraries by Bioanalyzer with high sensitivity DNA kit (Agilent Technologies, CA, USA).
Quantify the library using QuantiFluor dsDNA System and Quantus Fluorometer (Promega).

12-2. Sequencing of the library. We recommend the use of the Illumina platform with non-patterned flow cells such as HiSeq 2500 or MiSeq sequencer (Illumina) to suppress false-assignment among samples.

Primer sequences used in this protocol

PCR primers

SE PCR forward-primer	CAAGCAGAAGACGGCATACGAGAT
SE PCR reverse-primer	AATGATACGGCGACCACCGAGATCTACACTCGTCGGCAGCGTC

SE RT-primers (5’-3’) Index1~96, Index101~196

“n” in the sequence indicates unique molecular identifier.

Index 001	CAGAAGACGGCATACGAGATAACTTTAGTGACTGGAGTTCAGACGTGTGCTCTTCCGATCNNNNNNTTTTTTTTTTTTTTTTTTV
Index 002	CAGAAGACGGCATACGAGATCATAGAGTGTGACTGGAGTTCAGACGTGTGCTCTTCCGATCNNNNNNTTTTTTTTTTTTTTTTTTV
Index 003	CAGAAGACGGCATACGAGATTGCTGGAGGTGACTGGAGTTCAGACGTGTGCTCTTCCGATCNNNNNNTTTTTTTTTTTTTTTTTTV
Index 004	CAGAAGACGGCATACGAGATTTGGTGACGTGACTGGAGTTCAGACGTGTGCTCTTCCGATCNNNNNNTTTTTTTTTTTTTTTTTTV
Index 005	CAGAAGACGGCATACGAGATGTCCACGTGTGACTGGAGTTCAGACGTGTGCTCTTCCGATCNNNNNNTTTTTTTTTTTTTTTTTTV
Index 006	CAGAAGACGGCATACGAGATGAGACGGAGTGACTGGAGTTCAGACGTGTGCTCTTCCGATCNNNNNNTTTTTTTTTTTTTTTTTTV
Index 007	CAGAAGACGGCATACGAGATCCAGTATCGTGACTGGAGTTCAGACGTGTGCTCTTCCGATCNNNNNNTTTTTTTTTTTTTTTTTTV
Index 008	CAGAAGACGGCATACGAGATCCGATCTTGTGACTGGAGTTCAGACGTGTGCTCTTCCGATCNNNNNNTTTTTTTTTTTTTTTTTTV
Index 009	CAGAAGACGGCATACGAGATTGCGAGCCGTGACTGGAGTTCAGACGTGTGCTCTTCCGATCNNNNNNTTTTTTTTTTTTTTTTTTV
Index 010	CAGAAGACGGCATACGAGATATTCGTTAGTGACTGGAGTTCAGACGTGTGCTCTTCCGATCNNNNNNTTTTTTTTTTTTTTTTTTV
Index 011	CAGAAGACGGCATACGAGATTATTCGTAGTGACTGGAGTTCAGACGTGTGCTCTTCCGATCNNNNNNTTTTTTTTTTTTTTTTTTV
Index 012	CAGAAGACGGCATACGAGATCGGCCCAGGTGACTGGAGTTCAGACGTGTGCTCTTCCGATCNNNNNNTTTTTTTTTTTTTTTTTTV
Index 013	CAGAAGACGGCATACGAGATAATTGGCGGTGACTGGAGTTCAGACGTGTGCTCTTCCGATCNNNNNNTTTTTTTTTTTTTTTTTTV
Index 014	CAGAAGACGGCATACGAGATCGCCAGGGGTGACTGGAGTTCAGACGTGTGCTCTTCCGATCNNNNNNTTTTTTTTTTTTTTTTTTV
Index 015	CAGAAGACGGCATACGAGATAGATTCCGGTGACTGGAGTTCAGACGTGTGCTCTTCCGATCNNNNNNTTTTTTTTTTTTTTTTTTV
Index 016	CAGAAGACGGCATACGAGATTATCGAGAGTGACTGGAGTTCAGACGTGTGCTCTTCCGATCNNNNNNTTTTTTTTTTTTTTTTTTV
Index 017	CAGAAGACGGCATACGAGATTCCATGACGTGACTGGAGTTCAGACGTGTGCTCTTCCGATCNNNNNNTTTTTTTTTTTTTTTTTTV
Index 018	CAGAAGACGGCATACGAGATACCACATCGTGACTGGAGTTCAGACGTGTGCTCTTCCGATCNNNNNNTTTTTTTTTTTTTTTTTTV
Index 019	CAGAAGACGGCATACGAGATTAGTGTAAGTGACTGGAGTTCAGACGTGTGCTCTTCCGATCNNNNNNTTTTTTTTTTTTTTTTTTV
Index 020	CAGAAGACGGCATACGAGATCCGGGCGTGTGACTGGAGTTCAGACGTGTGCTCTTCCGATCNNNNNNTTTTTTTTTTTTTTTTTTV
Index 021	CAGAAGACGGCATACGAGATACCTGACGGTGACTGGAGTTCAGACGTGTGCTCTTCCGATCNNNNNNTTTTTTTTTTTTTTTTTTV
Index 022	CAGAAGACGGCATACGAGATCTAGGCAGGTGACTGGAGTTCAGACGTGTGCTCTTCCGATCNNNNNNTTTTTTTTTTTTTTTTTTV
Index 023	CAGAAGACGGCATACGAGATGGAACGCCGTGACTGGAGTTCAGACGTGTGCTCTTCCGATCNNNNNNTTTTTTTTTTTTTTTTTTV
Index 024	CAGAAGACGGCATACGAGATGGCATTTTGTGACTGGAGTTCAGACGTGTGCTCTTCCGATCNNNNNNTTTTTTTTTTTTTTTTTTV
Index 025	CAGAAGACGGCATACGAGATCGTGGATAGTGACTGGAGTTCAGACGTGTGCTCTTCCGATCNNNNNNTTTTTTTTTTTTTTTTTTV
Index 026	CAGAAGACGGCATACGAGATCCGACATGGTGACTGGAGTTCAGACGTGTGCTCTTCCGATCNNNNNNTTTTTTTTTTTTTTTTTTV
Index 027	CAGAAGACGGCATACGAGATTCCGCTTAGTGACTGGAGTTCAGACGTGTGCTCTTCCGATCNNNNNNTTTTTTTTTTTTTTTTTTV
Index 028	CAGAAGACGGCATACGAGATATTGGAGGGTGACTGGAGTTCAGACGTGTGCTCTTCCGATCNNNNNNTTTTTTTTTTTTTTTTTTV

Index 029	CAGAAGACGGCATACGAGATTTGACAGCGTGACTGGAGTTCAGACGTGTGCTCTTCCGATCNNNNNNTTTTTTTTTTTTTTTTTTV
Index 030	CAGAAGACGGCATACGAGATCCCAATCGGTGACTGGAGTTCAGACGTGTGCTCTTCCGATCNNNNNNTTTTTTTTTTTTTTTTTTV
Index 031	CAGAAGACGGCATACGAGATTAGAAGCCGTGACTGGAGTTCAGACGTGTGCTCTTCCGATCNNNNNNTTTTTTTTTTTTTTTTTTV
Index 032	CAGAAGACGGCATACGAGATAGAAGTGAGTGACTGGAGTTCAGACGTGTGCTCTTCCGATCNNNNNNTTTTTTTTTTTTTTTTTTV
Index 033	CAGAAGACGGCATACGAGATACATCATAGTGACTGGAGTTCAGACGTGTGCTCTTCCGATCNNNNNNTTTTTTTTTTTTTTTTTTV
Index 034	CAGAAGACGGCATACGAGATGGTTAAAAGTGACTGGAGTTCAGACGTGTGCTCTTCCGATCNNNNNNTTTTTTTTTTTTTTTTTTV
Index 035	CAGAAGACGGCATACGAGATCAGTGGCCGTGACTGGAGTTCAGACGTGTGCTCTTCCGATCNNNNNNTTTTTTTTTTTTTTTTTTV
Index 036	CAGAAGACGGCATACGAGATATTTAGGCGTGACTGGAGTTCAGACGTGTGCTCTTCCGATCNNNNNNTTTTTTTTTTTTTTTTTTV
Index 037	CAGAAGACGGCATACGAGATGAGGGCCCGTGACTGGAGTTCAGACGTGTGCTCTTCCGATCNNNNNNTTTTTTTTTTTTTTTTTTV
Index 038	CAGAAGACGGCATACGAGATCTCGTATTGTGACTGGAGTTCAGACGTGTGCTCTTCCGATCNNNNNNTTTTTTTTTTTTTTTTTTV
Index 039	CAGAAGACGGCATACGAGATGGATCGTGGTGACTGGAGTTCAGACGTGTGCTCTTCCGATCNNNNNNTTTTTTTTTTTTTTTTTTV
Index 040	CAGAAGACGGCATACGAGATCACTAATAGTGACTGGAGTTCAGACGTGTGCTCTTCCGATCNNNNNNTTTTTTTTTTTTTTTTTTV
Index 041	CAGAAGACGGCATACGAGATCCTTCGCTGTGACTGGAGTTCAGACGTGTGCTCTTCCGATCNNNNNNTTTTTTTTTTTTTTTTTTV
Index 042	CAGAAGACGGCATACGAGATAGATGGTCGTGACTGGAGTTCAGACGTGTGCTCTTCCGATCNNNNNNTTTTTTTTTTTTTTTTTTV
Index 043	CAGAAGACGGCATACGAGATGATAGGTAGTGACTGGAGTTCAGACGTGTGCTCTTCCGATCNNNNNNTTTTTTTTTTTTTTTTTTV
Index 044	CAGAAGACGGCATACGAGATAGCCGCTAGTGACTGGAGTTCAGACGTGTGCTCTTCCGATCNNNNNNTTTTTTTTTTTTTTTTTTV
Index 045	CAGAAGACGGCATACGAGATCTTTGTCAGTGACTGGAGTTCAGACGTGTGCTCTTCCGATCNNNNNNTTTTTTTTTTTTTTTTTTV
Index 046	CAGAAGACGGCATACGAGATACGCCACTGTGACTGGAGTTCAGACGTGTGCTCTTCCGATCNNNNNNTTTTTTTTTTTTTTTTTTV
Index 047	CAGAAGACGGCATACGAGATTAGACTGTGTGACTGGAGTTCAGACGTGTGCTCTTCCGATCNNNNNNTTTTTTTTTTTTTTTTTTV
Index 048	CAGAAGACGGCATACGAGATGTTCTCAAGTGACTGGAGTTCAGACGTGTGCTCTTCCGATCNNNNNNTTTTTTTTTTTTTTTTTTV
Index 049	CAGAAGACGGCATACGAGATAGCGTGACGTGACTGGAGTTCAGACGTGTGCTCTTCCGATCNNNNNNTTTTTTTTTTTTTTTTTTV
Index 050	CAGAAGACGGCATACGAGATGCGTCTACGTGACTGGAGTTCAGACGTGTGCTCTTCCGATCNNNNNNTTTTTTTTTTTTTTTTTTV
Index 051	CAGAAGACGGCATACGAGATTTACCAGGGTGACTGGAGTTCAGACGTGTGCTCTTCCGATCNNNNNNTTTTTTTTTTTTTTTTTTV
Index 052	CAGAAGACGGCATACGAGATGCCAGTTGGTGACTGGAGTTCAGACGTGTGCTCTTCCGATCNNNNNNTTTTTTTTTTTTTTTTTTV
Index 053	CAGAAGACGGCATACGAGATGGTCACGGGTGACTGGAGTTCAGACGTGTGCTCTTCCGATCNNNNNNTTTTTTTTTTTTTTTTTTV
Index 054	CAGAAGACGGCATACGAGATGACAAGATGTGACTGGAGTTCAGACGTGTGCTCTTCCGATCNNNNNNTTTTTTTTTTTTTTTTTTV
Index 055	CAGAAGACGGCATACGAGATGCCAGAAAGTGACTGGAGTTCAGACGTGTGCTCTTCCGATCNNNNNNTTTTTTTTTTTTTTTTTTV
Index 056	CAGAAGACGGCATACGAGATACAGAGGCGTGACTGGAGTTCAGACGTGTGCTCTTCCGATCNNNNNNTTTTTTTTTTTTTTTTTTV
Index 057	CAGAAGACGGCATACGAGATCGAGAGTCGTGACTGGAGTTCAGACGTGTGCTCTTCCGATCNNNNNNTTTTTTTTTTTTTTTTTTV
Index 058	CAGAAGACGGCATACGAGATCTCACGTAGTGACTGGAGTTCAGACGTGTGCTCTTCCGATCNNNNNNTTTTTTTTTTTTTTTTTTV
Index 059	CAGAAGACGGCATACGAGATCTTATTACGTGACTGGAGTTCAGACGTGTGCTCTTCCGATCNNNNNNTTTTTTTTTTTTTTTTTTV
Index 060	CAGAAGACGGCATACGAGATCGCTATTTGTGACTGGAGTTCAGACGTGTGCTCTTCCGATCNNNNNNTTTTTTTTTTTTTTTTTTV
Index 061	CAGAAGACGGCATACGAGATGTTGTCCTGTGACTGGAGTTCAGACGTGTGCTCTTCCGATCNNNNNNTTTTTTTTTTTTTTTTTTV
Index 062	CAGAAGACGGCATACGAGATTCAACTCTGTGACTGGAGTTCAGACGTGTGCTCTTCCGATCNNNNNNTTTTTTTTTTTTTTTTTTV
Index 063	CAGAAGACGGCATACGAGATATACTTACGTGACTGGAGTTCAGACGTGTGCTCTTCCGATCNNNNNNTTTTTTTTTTTTTTTTTTV
Index 064	CAGAAGACGGCATACGAGATTGTGTGGCGTGACTGGAGTTCAGACGTGTGCTCTTCCGATCNNNNNNTTTTTTTTTTTTTTTTTTV
Index 065	CAGAAGACGGCATACGAGATCGACTCTTGTGACTGGAGTTCAGACGTGTGCTCTTCCGATCNNNNNNTTTTTTTTTTTTTTTTTTV
Index 066	CAGAAGACGGCATACGAGATTCGCTCGCGTGACTGGAGTTCAGACGTGTGCTCTTCCGATCNNNNNNTTTTTTTTTTTTTTTTTTV

Index 067	CAGAAGACGGCATACGAGATACACGAACGTGACTGGAGTTCAGACGTGTGCTCTTCCGATCNNNNNNTTTTTTTTTTTTTTTTTV
Index 068	CAGAAGACGGCATACGAGATTTAAATGCGTGACTGGAGTTCAGACGTGTGCTCTTCCGATCNNNNNNTTTTTTTTTTTTTTTTTV
Index 069	CAGAAGACGGCATACGAGATCCTCGGCGGTGACTGGAGTTCAGACGTGTGCTCTTCCGATCNNNNNNTTTTTTTTTTTTTTTTTV
Index 070	CAGAAGACGGCATACGAGATTATATTAGGTGACTGGAGTTCAGACGTGTGCTCTTCCGATCNNNNNNTTTTTTTTTTTTTTTTTV
Index 071	CAGAAGACGGCATACGAGATGCTGGCTTGTGACTGGAGTTCAGACGTGTGCTCTTCCGATCNNNNNNTTTTTTTTTTTTTTTTTV
Index 072	CAGAAGACGGCATACGAGATTGGATGTAGTGACTGGAGTTCAGACGTGTGCTCTTCCGATCNNNNNNTTTTTTTTTTTTTTTTTV
Index 073	CAGAAGACGGCATACGAGATTTTAGCACGTGACTGGAGTTCAGACGTGTGCTCTTCCGATCNNNNNNTTTTTTTTTTTTTTTTTV
Index 074	CAGAAGACGGCATACGAGATCAGTACGGGTGACTGGAGTTCAGACGTGTGCTCTTCCGATCNNNNNNTTTTTTTTTTTTTTTTTV
Index 075	CAGAAGACGGCATACGAGATATCCTCCAGTGACTGGAGTTCAGACGTGTGCTCTTCCGATCNNNNNNTTTTTTTTTTTTTTTTTV
Index 076	CAGAAGACGGCATACGAGATGTCACTACGTGACTGGAGTTCAGACGTGTGCTCTTCCGATCNNNNNNTTTTTTTTTTTTTTTTTV
Index 077	CAGAAGACGGCATACGAGATTGGTACTCGTGACTGGAGTTCAGACGTGTGCTCTTCCGATCNNNNNNTTTTTTTTTTTTTTTTTV
Index 078	CAGAAGACGGCATACGAGATCCGAGTTAGTGACTGGAGTTCAGACGTGTGCTCTTCCGATCNNNNNNTTTTTTTTTTTTTTTTTV
Index 079	CAGAAGACGGCATACGAGATGCTCTGCCGTGACTGGAGTTCAGACGTGTGCTCTTCCGATCNNNNNNTTTTTTTTTTTTTTTTTV
Index 080	CAGAAGACGGCATACGAGATGCCTCGATGTGACTGGAGTTCAGACGTGTGCTCTTCCGATCNNNNNNTTTTTTTTTTTTTTTTTV
Index 081	CAGAAGACGGCATACGAGATTATCTCTCGTGACTGGAGTTCAGACGTGTGCTCTTCCGATCNNNNNNTTTTTTTTTTTTTTTTTV
Index 082	CAGAAGACGGCATACGAGATGCTGGTAAGTGACTGGAGTTCAGACGTGTGCTCTTCCGATCNNNNNNTTTTTTTTTTTTTTTTTV
Index 083	CAGAAGACGGCATACGAGATCAGGTGCTGTGACTGGAGTTCAGACGTGTGCTCTTCCGATCNNNNNNTTTTTTTTTTTTTTTTTV
Index 084	CAGAAGACGGCATACGAGATGAACCAGCGTGACTGGAGTTCAGACGTGTGCTCTTCCGATCNNNNNNTTTTTTTTTTTTTTTTTV
Index 085	CAGAAGACGGCATACGAGATGGTGCATTGTGACTGGAGTTCAGACGTGTGCTCTTCCGATCNNNNNNTTTTTTTTTTTTTTTTTV
Index 086	CAGAAGACGGCATACGAGATCTCGCTCAGTGACTGGAGTTCAGACGTGTGCTCTTCCGATCNNNNNNTTTTTTTTTTTTTTTTTV
Index 087	CAGAAGACGGCATACGAGATTGGAAGAGGTGACTGGAGTTCAGACGTGTGCTCTTCCGATCNNNNNNTTTTTTTTTTTTTTTTTV
Index 088	CAGAAGACGGCATACGAGATAATCGGGCGTGACTGGAGTTCAGACGTGTGCTCTTCCGATCNNNNNNTTTTTTTTTTTTTTTTTV
Index 089	CAGAAGACGGCATACGAGATATGGTTTCGTGACTGGAGTTCAGACGTGTGCTCTTCCGATCNNNNNNTTTTTTTTTTTTTTTTTV
Index 090	CAGAAGACGGCATACGAGATGCAGACCAGTGACTGGAGTTCAGACGTGTGCTCTTCCGATCNNNNNNTTTTTTTTTTTTTTTTTV
Index 091	CAGAAGACGGCATACGAGATGCACACTTGTGACTGGAGTTCAGACGTGTGCTCTTCCGATCNNNNNNTTTTTTTTTTTTTTTTTV
Index 092	CAGAAGACGGCATACGAGATAAGAGTTCGTGACTGGAGTTCAGACGTGTGCTCTTCCGATCNNNNNNTTTTTTTTTTTTTTTTTV
Index 093	CAGAAGACGGCATACGAGATCATTTATGGTGACTGGAGTTCAGACGTGTGCTCTTCCGATCNNNNNNTTTTTTTTTTTTTTTTTV
Index 094	CAGAAGACGGCATACGAGATCTGATGAGGTGACTGGAGTTCAGACGTGTGCTCTTCCGATCNNNNNNTTTTTTTTTTTTTTTTTV
Index 095	CAGAAGACGGCATACGAGATATAGAGAGGTGACTGGAGTTCAGACGTGTGCTCTTCCGATCNNNNNNTTTTTTTTTTTTTTTTTV
Index 096	CAGAAGACGGCATACGAGATGGAGGTATGTGACTGGAGTTCAGACGTGTGCTCTTCCGATCNNNNNNTTTTTTTTTTTTTTTTTV

Index 101	CAGAAGACGGCATACGAGATATCGTTGTGTGACTGGAGTTCAGACGTGTGCTCTTCCGATCNNNNNNTTTTTTTTTTTTTTTTTV
Index 102	CAGAAGACGGCATACGAGATTATACACAGTGACTGGAGTTCAGACGTGTGCTCTTCCGATCNNNNNNTTTTTTTTTTTTTTTTTV
Index 103	CAGAAGACGGCATACGAGATCCAGGGCCGTGACTGGAGTTCAGACGTGTGCTCTTCCGATCNNNNNNTTTTTTTTTTTTTTTTTV
Index 104	CAGAAGACGGCATACGAGATAAAGGAGCGTGACTGGAGTTCAGACGTGTGCTCTTCCGATCNNNNNNTTTTTTTTTTTTTTTTTV
Index 105	CAGAAGACGGCATACGAGATAAACTCCTGTGACTGGAGTTCAGACGTGTGCTCTTCCGATCNNNNNNTTTTTTTTTTTTTTTTTV
Index 106	CAGAAGACGGCATACGAGATCCACCGGGGTGACTGGAGTTCAGACGTGTGCTCTTCCGATCNNNNNNTTTTTTTTTTTTTTTTTV
Index 107	CAGAAGACGGCATACGAGATGTATTAGAGTGACTGGAGTTCAGACGTGTGCTCTTCCGATCNNNNNNTTTTTTTTTTTTTTTTTV

Index 108	CAGAAGACGGCATACGAGATACAACCATGTGACTGGAGTTCAGACGTGTGCTCTTCCGATCNNNNNNTTTTTTTTTTTTTTTTTTV
Index 109	CAGAAGACGGCATACGAGATTGGGTTCCGTGACTGGAGTTCAGACGTGTGCTCTTCCGATCNNNNNNTTTTTTTTTTTTTTTTTTV
Index 110	CAGAAGACGGCATACGAGATCGGCATAAGTGACTGGAGTTCAGACGTGTGCTCTTCCGATCNNNNNNTTTTTTTTTTTTTTTTTTV
Index 111	CAGAAGACGGCATACGAGATCTCCTTTAGTGACTGGAGTTCAGACGTGTGCTCTTCCGATCNNNNNNTTTTTTTTTTTTTTTTTTV
Index 112	CAGAAGACGGCATACGAGATGATACTAAGTGACTGGAGTTCAGACGTGTGCTCTTCCGATCNNNNNNTTTTTTTTTTTTTTTTTTV
Index 113	CAGAAGACGGCATACGAGATCCTAATTCGTGACTGGAGTTCAGACGTGTGCTCTTCCGATCNNNNNNTTTTTTTTTTTTTTTTTTV
Index 114	CAGAAGACGGCATACGAGATTCACTACGGTGACTGGAGTTCAGACGTGTGCTCTTCCGATCNNNNNNTTTTTTTTTTTTTTTTTTV
Index 115	CAGAAGACGGCATACGAGATTAGCGTGCGTGACTGGAGTTCAGACGTGTGCTCTTCCGATCNNNNNNTTTTTTTTTTTTTTTTTTV
Index 116	CAGAAGACGGCATACGAGATCTCCAAGCGTGACTGGAGTTCAGACGTGTGCTCTTCCGATCNNNNNNTTTTTTTTTTTTTTTTTTV
Index 117	CAGAAGACGGCATACGAGATCTTCATATGTGACTGGAGTTCAGACGTGTGCTCTTCCGATCNNNNNNTTTTTTTTTTTTTTTTTTV
Index 118	CAGAAGACGGCATACGAGATTGGCGCCGGTGACTGGAGTTCAGACGTGTGCTCTTCCGATCNNNNNNTTTTTTTTTTTTTTTTTTV
Index 119	CAGAAGACGGCATACGAGATCCACATCTGTGACTGGAGTTCAGACGTGTGCTCTTCCGATCNNNNNNTTTTTTTTTTTTTTTTTTV
Index 120	CAGAAGACGGCATACGAGATCGAGACCTGTGACTGGAGTTCAGACGTGTGCTCTTCCGATCNNNNNNTTTTTTTTTTTTTTTTTTV
Index 121	CAGAAGACGGCATACGAGATGTGAGGCAGTGACTGGAGTTCAGACGTGTGCTCTTCCGATCNNNNNNTTTTTTTTTTTTTTTTTTV
Index 122	CAGAAGACGGCATACGAGATTCTCGTGTGTGACTGGAGTTCAGACGTGTGCTCTTCCGATCNNNNNNTTTTTTTTTTTTTTTTTTV
Index 123	CAGAAGACGGCATACGAGATTTACGATGTGACTGGAGTTCAGACGTGTGCTCTTCCGATCNNNNNNTTTTTTTTTTTTTTTTTTV
Index 124	CAGAAGACGGCATACGAGATATAACGTCGTGACTGGAGTTCAGACGTGTGCTCTTCCGATCNNNNNNTTTTTTTTTTTTTTTTTTV
Index 125	CAGAAGACGGCATACGAGATACACGCTGGTGACTGGAGTTCAGACGTGTGCTCTTCCGATCNNNNNNTTTTTTTTTTTTTTTTTTV
Index 126	CAGAAGACGGCATACGAGATTTAAGACCGTGACTGGAGTTCAGACGTGTGCTCTTCCGATCNNNNNNTTTTTTTTTTTTTTTTTTV
Index 127	CAGAAGACGGCATACGAGATTTCCCATCGTGACTGGAGTTCAGACGTGTGCTCTTCCGATCNNNNNNTTTTTTTTTTTTTTTTTTV
Index 128	CAGAAGACGGCATACGAGATGTGACCCCGTGACTGGAGTTCAGACGTGTGCTCTTCCGATCNNNNNNTTTTTTTTTTTTTTTTTTV
Index 129	CAGAAGACGGCATACGAGATGACCGCGCGTGACTGGAGTTCAGACGTGTGCTCTTCCGATCNNNNNNTTTTTTTTTTTTTTTTTTV
Index 130	CAGAAGACGGCATACGAGATGTCGCAAAGTGACTGGAGTTCAGACGTGTGCTCTTCCGATCNNNNNNTTTTTTTTTTTTTTTTTTV
Index 131	CAGAAGACGGCATACGAGATGCGATCAAGTGACTGGAGTTCAGACGTGTGCTCTTCCGATCNNNNNNTTTTTTTTTTTTTTTTTTV
Index 132	CAGAAGACGGCATACGAGATTAGGCTAGGTGACTGGAGTTCAGACGTGTGCTCTTCCGATCNNNNNNTTTTTTTTTTTTTTTTTTV
Index 133	CAGAAGACGGCATACGAGATGTCATATAGTGACTGGAGTTCAGACGTGTGCTCTTCCGATCNNNNNNTTTTTTTTTTTTTTTTTTV
Index 134	CAGAAGACGGCATACGAGATAGTGTATCGTGACTGGAGTTCAGACGTGTGCTCTTCCGATCNNNNNNTTTTTTTTTTTTTTTTTTV
Index 135	CAGAAGACGGCATACGAGATGTCTTGTGGTGACTGGAGTTCAGACGTGTGCTCTTCCGATCNNNNNNTTTTTTTTTTTTTTTTTTV
Index 136	CAGAAGACGGCATACGAGATGACGTTATGTGACTGGAGTTCAGACGTGTGCTCTTCCGATCNNNNNNTTTTTTTTTTTTTTTTTTV
Index 137	CAGAAGACGGCATACGAGATCATGATCCGTGACTGGAGTTCAGACGTGTGCTCTTCCGATCNNNNNNTTTTTTTTTTTTTTTTTTV
Index 138	CAGAAGACGGCATACGAGATCAGCAAGTGTGACTGGAGTTCAGACGTGTGCTCTTCCGATCNNNNNNTTTTTTTTTTTTTTTTTTV
Index 139	CAGAAGACGGCATACGAGATGGCTTAATGTGACTGGAGTTCAGACGTGTGCTCTTCCGATCNNNNNNTTTTTTTTTTTTTTTTTTV
Index 140	CAGAAGACGGCATACGAGATAACGACGAGTGACTGGAGTTCAGACGTGTGCTCTTCCGATCNNNNNNTTTTTTTTTTTTTTTTTTV
Index 141	CAGAAGACGGCATACGAGATGCAAGCGGGTGACTGGAGTTCAGACGTGTGCTCTTCCGATCNNNNNNTTTTTTTTTTTTTTTTTTV
Index 142	CAGAAGACGGCATACGAGATAAGAGCGTGTGACTGGAGTTCAGACGTGTGCTCTTCCGATCNNNNNNTTTTTTTTTTTTTTTTTTV
Index 143	CAGAAGACGGCATACGAGATAGGCTCGAGTGACTGGAGTTCAGACGTGTGCTCTTCCGATCNNNNNNTTTTTTTTTTTTTTTTTTV
Index 144	CAGAAGACGGCATACGAGATGCAGTCGTGTGACTGGAGTTCAGACGTGTGCTCTTCCGATCNNNNNNTTTTTTTTTTTTTTTTTTV
Index 145	CAGAAGACGGCATACGAGATTTCACCTTGTGACTGGAGTTCAGACGTGTGCTCTTCCGATCNNNNNNTTTTTTTTTTTTTTTTTTV

Index 146	CAGAAGACGGCATACGAGATTCCTGTCCGTGACTGGAGTTCAGACGTGTGCTCTTCCGATCNNNNNNTTTTTTTTTTTTTTTTTTV
Index 147	CAGAAGACGGCATACGAGATACCCCGTAGTGACTGGAGTTCAGACGTGTGCTCTTCCGATCNNNNNNTTTTTTTTTTTTTTTTTTV
Index 148	CAGAAGACGGCATACGAGATAAAGAGTTGTGACTGGAGTTCAGACGTGTGCTCTTCCGATCNNNNNNTTTTTTTTTTTTTTTTTTV
Index 149	CAGAAGACGGCATACGAGATGATGAAATGTGACTGGAGTTCAGACGTGTGCTCTTCCGATCNNNNNNTTTTTTTTTTTTTTTTTTV
Index 150	CAGAAGACGGCATACGAGATGACCATAAGTGACTGGAGTTCAGACGTGTGCTCTTCCGATCNNNNNNTTTTTTTTTTTTTTTTTTV
Index 151	CAGAAGACGGCATACGAGATAGCAAGTAGTGACTGGAGTTCAGACGTGTGCTCTTCCGATCNNNNNNTTTTTTTTTTTTTTTTTTV
Index 152	CAGAAGACGGCATACGAGATAAACTGAGGTGACTGGAGTTCAGACGTGTGCTCTTCCGATCNNNNNNTTTTTTTTTTTTTTTTTTV
Index 153	CAGAAGACGGCATACGAGATGAGCGATAGTGACTGGAGTTCAGACGTGTGCTCTTCCGATCNNNNNNTTTTTTTTTTTTTTTTTTV
Index 154	CAGAAGACGGCATACGAGATGTTCTTCGGTGACTGGAGTTCAGACGTGTGCTCTTCCGATCNNNNNNTTTTTTTTTTTTTTTTTTV
Index 155	CAGAAGACGGCATACGAGATCCTGAGACGTGACTGGAGTTCAGACGTGTGCTCTTCCGATCNNNNNNTTTTTTTTTTTTTTTTTTV
Index 156	CAGAAGACGGCATACGAGATAGGTGAACGTGACTGGAGTTCAGACGTGTGCTCTTCCGATCNNNNNNTTTTTTTTTTTTTTTTTTV
Index 157	CAGAAGACGGCATACGAGATCACATCTGGTGACTGGAGTTCAGACGTGTGCTCTTCCGATCNNNNNNTTTTTTTTTTTTTTTTTTV
Index 158	CAGAAGACGGCATACGAGATTCTCAATCGTGACTGGAGTTCAGACGTGTGCTCTTCCGATCNNNNNNTTTTTTTTTTTTTTTTTTV
Index 159	CAGAAGACGGCATACGAGATCACAACGTGTGACTGGAGTTCAGACGTGTGCTCTTCCGATCNNNNNNTTTTTTTTTTTTTTTTTTV
Index 160	CAGAAGACGGCATACGAGATCACATACAGTGACTGGAGTTCAGACGTGTGCTCTTCCGATCNNNNNNTTTTTTTTTTTTTTTTTTV
Index 161	CAGAAGACGGCATACGAGATAGTTATCCGTGACTGGAGTTCAGACGTGTGCTCTTCCGATCNNNNNNTTTTTTTTTTTTTTTTTTV
Index 162	CAGAAGACGGCATACGAGATAAGCCTTTGTGACTGGAGTTCAGACGTGTGCTCTTCCGATCNNNNNNTTTTTTTTTTTTTTTTTTV
Index 163	CAGAAGACGGCATACGAGATTAAAGCCGGTGACTGGAGTTCAGACGTGTGCTCTTCCGATCNNNNNNTTTTTTTTTTTTTTTTTTV
Index 164	CAGAAGACGGCATACGAGATTCTAGACTGTGACTGGAGTTCAGACGTGTGCTCTTCCGATCNNNNNNTTTTTTTTTTTTTTTTTTV
Index 165	CAGAAGACGGCATACGAGATAGTCCACAGTGACTGGAGTTCAGACGTGTGCTCTTCCGATCNNNNNNTTTTTTTTTTTTTTTTTTV
Index 166	CAGAAGACGGCATACGAGATGCACGTCGGTGACTGGAGTTCAGACGTGTGCTCTTCCGATCNNNNNNTTTTTTTTTTTTTTTTTTV
Index 167	CAGAAGACGGCATACGAGATGGCCTTGGGTGACTGGAGTTCAGACGTGTGCTCTTCCGATCNNNNNNTTTTTTTTTTTTTTTTTTV
Index 168	CAGAAGACGGCATACGAGATTTGTCCGTGTGACTGGAGTTCAGACGTGTGCTCTTCCGATCNNNNNNTTTTTTTTTTTTTTTTTTV
Index 169	CAGAAGACGGCATACGAGATGATGCACGGTGACTGGAGTTCAGACGTGTGCTCTTCCGATCNNNNNNTTTTTTTTTTTTTTTTTTV
Index 170	CAGAAGACGGCATACGAGATGACGTGCCGTGACTGGAGTTCAGACGTGTGCTCTTCCGATCNNNNNNTTTTTTTTTTTTTTTTTTV
Index 171	CAGAAGACGGCATACGAGATTTAGGTACGTGACTGGAGTTCAGACGTGTGCTCTTCCGATCNNNNNNTTTTTTTTTTTTTTTTTTV
Index 172	CAGAAGACGGCATACGAGATACTCTATGGTGACTGGAGTTCAGACGTGTGCTCTTCCGATCNNNNNNTTTTTTTTTTTTTTTTTTV
Index 173	CAGAAGACGGCATACGAGATGGATTTTCGTGACTGGAGTTCAGACGTGTGCTCTTCCGATCNNNNNNTTTTTTTTTTTTTTTTTTV
Index 174	CAGAAGACGGCATACGAGATGGATAGACGTGACTGGAGTTCAGACGTGTGCTCTTCCGATCNNNNNNTTTTTTTTTTTTTTTTTTV
Index 175	CAGAAGACGGCATACGAGATCTCTTAAAGTGACTGGAGTTCAGACGTGTGCTCTTCCGATCNNNNNNTTTTTTTTTTTTTTTTTTV
Index 176	CAGAAGACGGCATACGAGATGCATAGCTGTGACTGGAGTTCAGACGTGTGCTCTTCCGATCNNNNNNTTTTTTTTTTTTTTTTTTV
Index 177	CAGAAGACGGCATACGAGATTGCCCAAGGTGACTGGAGTTCAGACGTGTGCTCTTCCGATCNNNNNNTTTTTTTTTTTTTTTTTTV
Index 178	CAGAAGACGGCATACGAGATCGTTACGAGTGACTGGAGTTCAGACGTGTGCTCTTCCGATCNNNNNNTTTTTTTTTTTTTTTTTTV
Index 179	CAGAAGACGGCATACGAGATAAGGTGAAGTGACTGGAGTTCAGACGTGTGCTCTTCCGATCNNNNNNTTTTTTTTTTTTTTTTTTV
Index 180	CAGAAGACGGCATACGAGATTTCTAACAGTGACTGGAGTTCAGACGTGTGCTCTTCCGATCNNNNNNTTTTTTTTTTTTTTTTTTV
Index 181	CAGAAGACGGCATACGAGATGTGCAACCGTGACTGGAGTTCAGACGTGTGCTCTTCCGATCNNNNNNTTTTTTTTTTTTTTTTTTV
Index 182	CAGAAGACGGCATACGAGATACTGCAGCGTGACTGGAGTTCAGACGTGTGCTCTTCCGATCNNNNNNTTTTTTTTTTTTTTTTTTV
Index 183	CAGAAGACGGCATACGAGATGGTCGATCGTGACTGGAGTTCAGACGTGTGCTCTTCCGATCNNNNNNTTTTTTTTTTTTTTTTTTV

Index 184	CAGAAGACGGCATACGAGATGTGTGATCGTGACTGGAGTTCAGACGTGTGCTCTTCCGATCNNNNNNTTTTTTTTTTTTTTTTTTV
Index 185	CAGAAGACGGCATACGAGATCAGCCTCGGTGACTGGAGTTCAGACGTGTGCTCTTCCGATCNNNNNNTTTTTTTTTTTTTTTTTTV
Index 186	CAGAAGACGGCATACGAGATTGACTAGAGTGACTGGAGTTCAGACGTGTGCTCTTCCGATCNNNNNNTTTTTTTTTTTTTTTTTTV
Index 187	CAGAAGACGGCATACGAGATAACAGCACGTGACTGGAGTTCAGACGTGTGCTCTTCCGATCNNNNNNTTTTTTTTTTTTTTTTTTV
Index 188	CAGAAGACGGCATACGAGATTGCGATAGGTGACTGGAGTTCAGACGTGTGCTCTTCCGATCNNNNNNTTTTTTTTTTTTTTTTTTV
Index 189	CAGAAGACGGCATACGAGATCCAGATAAGTGACTGGAGTTCAGACGTGTGCTCTTCCGATCNNNNNNTTTTTTTTTTTTTTTTTTV
Index 190	CAGAAGACGGCATACGAGATGGGATCTGGTGACTGGAGTTCAGACGTGTGCTCTTCCGATCNNNNNNTTTTTTTTTTTTTTTTTTV
Index 191	CAGAAGACGGCATACGAGATTATTTCGGGTGACTGGAGTTCAGACGTGTGCTCTTCCGATCNNNNNNTTTTTTTTTTTTTTTTTTV
Index 192	CAGAAGACGGCATACGAGATAGCCGTAGGTGACTGGAGTTCAGACGTGTGCTCTTCCGATCNNNNNNTTTTTTTTTTTTTTTTTTV
Index 193	CAGAAGACGGCATACGAGATGTACCTTGGTGACTGGAGTTCAGACGTGTGCTCTTCCGATCNNNNNNTTTTTTTTTTTTTTTTTTV
Index 194	CAGAAGACGGCATACGAGATCCAAGTGCGTGACTGGAGTTCAGACGTGTGCTCTTCCGATCNNNNNNTTTTTTTTTTTTTTTTTTV
Index 195	CAGAAGACGGCATACGAGATTCTCCTTGGTGACTGGAGTTCAGACGTGTGCTCTTCCGATCNNNNNNTTTTTTTTTTTTTTTTTTV
Index 196	CAGAAGACGGCATACGAGATAGCTTCAAGTGACTGGAGTTCAGACGTGTGCTCTTCCGATCNNNNNNTTTTTTTTTTTTTTTTTTV

Supplementary note 2: R and python script for RNA-Seq analysis

# Formation of RSEM-output files for R analysis (make “Index%03d.genes.results” files) ----------------------

```
$BINDIR/batch_makeRSEMout_noid.sh ¥
        $OUTDIR ¥
        $FINALDIR ¥
        $BED
date
if [ ! -e $BASEDIR/data/count_reads.tsv ]; then
        FILES=`ls $BASEDIR/data/org/*.fastq.gz; ls $BASEDIR/data/preprocessed/*.fq`
        echo "### count reads"
        /opt/bio/work/python/fastxStat.py -t fastq -i $FILES > $BASEDIR/data/count_reads.tsv
        date
fi
if [ ! -e $FINALDIR/count_mapped_reads.tsv ]; then
        echo "### count mapped reads"
        FILES=`ls $FINALDIR/*.results`
        /opt/bio/work/python/parseRSEMresult.py -i $FILES >
$FINALDIR/count_mapped_reads.tsv
        date
fi
if [ ! -e $FINALDIR/merged_stat.tsv ]; then
        echo "### merge TSVs"
        cat $FINALDIR/count_mapped_reads.tsv |grep -v "^#"|    cut -f 1,4,5 | sed -e "s/.genes//"
> $FINALDIR/count_mapped_reads.shaped.tsv
        /opt/bio/work/python/mergeById.py -1 $BASEDIR/data/count_reads.tsv -c1 0 -s1 1 -2
$FINALDIR/count_mapped_reads.shaped.tsv -c2 0 -s2 0 > $FINALDIR/merged_stat.tsv
        date
fi
rm -f $OUTDIR/*.transcript.bam
```

# fastxStat.py (python) --

```
#!/usr/bin/env python
import sys, os
import gzip, bz2
from Bio import SeqIO
import numpy as np

headers = [
        "Sample name",
        "Total reads",
        "Total base",
        "Average length",
        "Median length",
        "Var of length",
        "Std of length",
        "Max length",
        "Min length"
]
def main():
        #=======================================
        # parse args
        #---------------------------------------
        import argparse
        parser = argparse.ArgumentParser(description='This script outputs information of
```

```
fasta(or fastq) file(s).')
	parser.add_argument("-i", metavar="fastq_file", dest="infiles", required=True, nargs="+",
help='support gz file')
	parser.add_argument("-t", metavar="type", dest="type", required=True, choices=["fasta",
"fastq"], help='fasta or fastq')
	args = parser.parse_args()

	print "¥t".join(headers)

	all_lList = []
	#========================================
	# iterate files
	#----------------------------------------
	for infile in args.infiles:
	fileName = os.path.basename(infile)
	  fileDir = os.path.dirname(infile)
	fileId, fileExt = os.path.splitext(fileName)
	#========================================
	                # open file
	#----------------------------------------
	              FHR = None
	          if fileExt == ".gz":
	                              FHR = gzip.open(infile, "rb")
	        elif fileExt == ".bz2":
	                              FHR = bz2.BZ2File(infile, "r")
	                    else:
	                              FHR = open(infile, "rU")
	#========================================
	              # iterate reads
	#----------------------------------------
	                 lList = []
	for record in SeqIO.parse(FHR, args.type):
	                              id = record.id
	                              seq = record.seq
	                              l = len(seq)
	                              lList.append(l)
	               FHR.close()
	        all_lList.extend(lList)
	        lList = np.array(lList)
	       outputRow(fileId, lList)
	all_lList = np.array(all_lList)
	outputRow("Sum", all_lList)
def outputRow(rowname, lList):
	if len(lList) > 0:
	                 out = []
	        out.append(rowname)
	        out.append(len(lList))
	      out.append(np.sum(lList))
	out.append(round(np.average(lList), 2))
	out.append(round(np.median(lList), 2))
	out.append(round(np.var(lList), 2))
	out.append(round(np.std(lList), 2))
	     out.append(np.amax(lList))
	     out.append(np.amin(lList))
	    print "¥t".join(map(str, out))
	else:
	print "¥t".join(map(str, [rowname, 0, 0, 0, 0, 0, 0, 0, 0]))

# Run as script
if __name__=="__main__":
	main()
```

parseRSEMresult.py (python) --

```
#!/usr/bin/env python
import sys, os, re
from subprocess import Popen, PIPE, check_call
import numpy as np

SAMTOOLS = "samtools"
```

```
def main():
			#==========================================
			# parse args
			#------------------------------------------
			import argparse
			parser = argparse.ArgumentParser(description='This script outputs information of bam
file(s).')
			parser.add_argument("-i", metavar="RSEM_result_file", dest="infiles", required=True,
nargs="+")
			args = parser.parse_args()
			#==========================================
			# iterate files
			#------------------------------------------
			c = 0
			for infile in args.infiles:
			fileName = os.path.basename(infile)
			  fileDir = os.path.dirname(infile)
			fileId, fileExt = os.path.splitext(fileName)
			#==========================================
					# open file
			 #------------------------------------------
			cmd = "cat " + infile + " | grep '^#'"
			p = Popen([cmd], shell=True, stdout=PIPE)
			#==========================================
				# iterate records
			 #------------------------------------------
				titles = ["sample name"]
					values = [fileId]
				   for row in p.stdout:
							row = row.rstrip() # chomp
							itemList = row.split('¥t')
							if len(itemList) < 1:
								continue

							title = re.sub("^#", "", itemList[0])
							titles.append(title)
							values.append(itemList[1])
					p.wait()
				  p.stdout.close()

					if c < 1:
							print "¥t".join(titles)
				print "¥t".join(values)

					c += 1
# Run as script
if __name__=="__main__":
			main()
```

mergeById.py (python) --

```
#!/usr/bin/env python
import os, sys, re
import numpy as np

def main():
			import argparse
			parser = argparse.ArgumentParser(description='')
			parser.add_argument("-i", metavar="input.tsv", dest="input", required=True, nargs="+")
			parser.add_argument("-s", metavar="num of skip row", dest="skip", required=True,
type=int)
			parser.add_argument("-c", metavar="id col index", dest="col", required=True, type=int)
			parser.add_argument("-v", metavar="value col index", dest="val", required=True,
type=int)
			args = parser.parse_args()

			id_map = {}
			file_num = len(args.input)
			file_names = [""]
```

```
file_index = 0
for infile in args.input:
fileName = os.path.basename(infile)
  fileDir = os.path.dirname(infile)
fileId, fileExt = os.path.splitext(fileName)

       file_names.append(fileId)

       FHR = open(infile, "rU")
        for i in range(args.skip):
                        FHR.next()
            for row in FHR:
                        row = row.rstrip() # chomp
                        itemList = row.split('¥t')
                        if len(itemList) < 1:
                                continue
                        id = itemList[args.col]
                        if not id_map.has_key(id):
                                id_map[id] = np.empty(file_num, dtype=object)
                        id_map[id][file_index] = str(itemList[args.val])

              FHR.close()
            file_index += 1
print "¥t".join(file_names)

for id in sorted(id_map.keys()):
              out = [id]
         for val in id_map[id]:
                        if val == None:
                                val = "."
                        out.append(val)
          print "¥t".join(out)

# map_2 = {}

# for infile in args.input2:
#      FHR = open(infile, "rU")
#      for i in range(args.skip2):
#                       FHR.next()
#            for row in FHR:
#                       row = row.rstrip() # chomp
#                       itemList = row.split('¥t')
#                       if len(itemList) < 1:
#                               continue
#                       id = itemList.pop(args.col2)
#                       if not map_2.has_key(id):
#                               map_2[id] = itemList
#                       # else:
#                       #       sys.exit("ERROR: ID duplication >> " + id)
#              FHR.close()

  # FHR = open(args.input1, "rU")
# for i in range(args.skip1):
#              FHR.next()
# for row in FHR:
#    row = row.rstrip() # chomp
#      itemList = row.split('¥t')
#         if len(itemList) < 1:
#                       continue

#      id = itemList[args.col1]
#       if map_2.has_key(id):
#                       itemList.extend(map_2[id])
#      print "¥t".join(itemList)
# FHR.close()
# Run as script
if __name__=="__main__":
        main()
```

# analysis using “Index%03d.genes.results” files ------------------------------------

```
# set parameters --------------------------------------------------------------------------------
dir.input <- "Pass of the input directory"
dir.output <- "Pass of the input directory"
dir.rawdata <- "Pass of the directory in which “Index%03d.genes.results” files were placed"

fn.description <- "Araport11_genes201606transcript_rep_ERCC_Virus7457_desc"
fn.sample.index <- "Sample-IndexPrimer.txt"
fn.sample.index.merged <- "Sample-IndexPrimer_ merged.txt"

merge.sample <- TRUE
exec.date <- "date of analysis"
species <- "Ath"

fn.rawcnt <- sprintf("%s-2_rawcnt_%s", exec.date, species)      # raw read count
fn.rpm <- sprintf("%s-3_rpm_%s", exec.date, species)             # rpm
fn.cvrd1 <- sprintf("%s-4_cvrd1_%s", exec.date, species)        # coverage     (percentage of sequence covered by
                                                                                more than 1 read within each gene)
fn.cvrd3 <- sprintf("%s-5_cvrd3_%s", exec.date, species)        # coverage (coverage by more than 3 reads)
fn.readall <- sprintf("%s-6_readall_%s", exec.date, species)    # number of all reads
fn.readfw <- sprintf("%s-7_readfw_%s", exec.date, species)     # number of forward reads
fn.fwpct <- sprintf("%s-8_fwpct_%s", exec.date, species)        # percentage of the forward reads against all reads
fn.rcsummary <- sprintf("%s-1_ReadCountSummary_%s.txt", exec.date, species)

#load sample attribute ------------------------------------------------------------------------
fn <- sprintf("%s/%s", dir.input, fn.sample.index)
at <- read.delim(fn, header=T, as.is=T)
rownames(at) <- sprintf("%s_%03d", at[,"library"], at[,"index"])

#load transcript description ------------------------------------------------------------------
fn <- sprintf("%s/%s", dir.input, fn.description)
load(fn)

#set data read row ----------------------------------------------------------------------------
drr <- des[,"NormalizationGroup"]=="data"
names(drr) <- rownames(des)

# save/load expression data table------------------------------------------------------------
rawcnt <- matrix(0, nrow=nrow(des), ncol=nrow(at))
colnames(rawcnt) <- rownames(at)
rownames(rawcnt) <- rownames(des)
cvrd1 <- rawcnt
cvrd3 <- rawcnt
readall <- rawcnt
readfw <- rawcnt
fwpct <- rawcnt
readcount <- matrix(0, nrow=nrow(at), ncol=4)
colnames(readcount) <- c("Total reads", "Unique sequences", "Mapped reads", "Mapped rate")

for(i in 1:nrow(at)){
  fn <- sprintf("%s/%s/%s/Index%03d.genes.results",
                          dir.input, dir.rawdata, at[i,"library"], at[i,"index"])
```

```
    tmp <- read.delim(fn, nrows=5, fill=T, header=F, as.is=T)
    readcount[i,] <- c(tmp[2,2], tmp[3,2], tmp[4,2], tmp[5,2])
    #readcount[i,] <- c(tmp[2,2], tmp[2,2], tmp[3,2], tmp[4,2])

    tmp <- read.delim(fn, as.is=T, comment.char="#")
    rawcnt[,i] <- tmp[,"expected_count"]
    if(is.element("coverage.depth..1.",colnames(tmp))){
        cvrd1[,i] <- tmp[,"coverage.depth..1."]
        cvrd3[,i] <- tmp[,"coverage.depth..3."]
    }
    if(is.element("mapped.reads.all.",colnames(tmp))){
        readall[,i] <- tmp[,"mapped.reads.all."]
        readfw[,i] <- tmp[,"mapped.reads.fw."]
        fwpct[,i] <- tmp[,"fw..."]
    }

# subtruction of indexing contamination-------------------------------------------------
ulib <- unique(at[,"library"])

rawcnttmp <- NULL
for(i in ulib){
    extmp <- rawcnt[, at[,"library"]==i]
    tmp <- rowSums(extmp)*0.0005
    extmp <- extmp-tmp
    rawcnttmp <- cbind(rawcnttmp, extmp)
}
rawcnttmp[rawcnttmp<0] <- 0

rawcnt <- rawcnttmp[,rownames(at)]
cvrd1[rawcnt==0] <- 0
cvrd3[rawcnt==0] <- 0
readall[rawcnt==0] <- 0
readfw[rawcnt==0] <- 0
fwpct[rawcnt==0] <- 0

# marge multi-fastq from 1 sample to 1 coloumn----------------------------------------
if(merge.sample){
    uni.sampleID <- unique(at[,"sampleID"])
    # at
    at.tmp <- NULL
    for(i in 1:length(uni.sampleID)){
        tmp <- at[at[,"sampleID"] == uni.sampleID[i], ]

        if(is.vector(tmp)){
            at.tmp <- rbind(at.tmp, tmp)
        } else {
            at.tmp <- rbind(at.tmp, tmp[1,])
        }
    }
    at.ori <- at
    at <- at.tmp
    write.table(at, file=sprintf("%s/%s", dir.input, fn.sample.index.merged), sep="¥t")
    at <- at.ori
```

```
  # rawcnt-------------------------------------------------------------------------------------
  tmp <- rawcnt
  tmp.fun <- function(x){
    x2 <- aggregate(x, by=list(at[,"sampleID"]), FUN=sum)
    x3 <- x2[,2]
    names(x3) <- x2[,1]
    return(x3)
  }
  tmp2 <- apply(tmp, 1, FUN=tmp.fun)
  tmp3 <- t(tmp2)
  tmp4 <- tmp3[,uni.sampleID]
  rawcnt <- tmp4
}

#calc. rpm-------------------------------------------------------------------------------------
rce <- rawcnt[des[,"NormalizationGroup"]=="data", ]
tmp <- colSums(rce)/10^6
rce2 <- t(t(rce)/tmp)
if(sum(des[,"NormalizationGroup"]=="data")==0){rce2<-NULL}

rcv <- rawcnt[des[,"NormalizationGroup"]=="virus", ]
tmp <- colSums(rce)/10^6
rcv2 <- t(t(rcv)/tmp)
if(sum(des[,"NormalizationGroup"]=="virus")==0){rcv2<-NULL}

rcb <- rawcnt[des[,"NormalizationGroup"]=="rRNA", ]
tmp <- colSums(rcb)/10^6
rcb2 <- t(t(rcb)/tmp)
if(sum(des[,"NormalizationGroup"]=="rRNA")==0){rcb2<-NULL}

rcc <- rawcnt[des[,"NormalizationGroup"]=="ercc", ]
tmp <- colSums(rcc)/10^6
rcc2 <- t(t(rcc)/tmp)
if(sum(des[,"NormalizationGroup"]=="ercc")==0){rcc2<-NULL}

rpm <- rbind(rce2, rcv2, rcb2, rcc2)
rpm[is.nan(rpm)] <- 0
rpm <- rpm[rownames(des),]

# save objects---------------------------------------------------------------------------------
save(rawcnt, file=sprintf("%s/%s", dir.input, fn.rawcnt))
save(rpm, file=sprintf("%s/%s", dir.input, fn.rpm))
save(cvrd1, file=sprintf("%s/%s", dir.input, fn.cvrd1))
save(cvrd3, file=sprintf("%s/%s", dir.input, fn.cvrd3))
save(readall, file=sprintf("%s/%s", dir.input, fn.readall))
save(readfw, file=sprintf("%s/%s", dir.input, fn.readfw))
save(fwpct, file=sprintf("%s/%s", dir.input, fn.fwpct))

write.table(cbind(readcount, at), file=sprintf("%s/%s", dir.output, fn.rcsummary))
```
